# Genotyping and Molecular Characterization of Classical Swine Fever Virus Isolated in China during 2016–2018

**DOI:** 10.3390/v13040664

**Published:** 2021-04-12

**Authors:** Madiha Fatima, Yuzi Luo, Li Zhang, Peng-Ying Wang, Hao Song, Yanhui Fu, Yongfeng Li, Yuan Sun, Su Li, Yun-Juan Bao, Hua-Ji Qiu

**Affiliations:** 1 State Key Laboratory of Veterinary Biotechnology, Harbin Veterinary Research Institute, Chinese Academy of Agricultural Sciences, 678 Haping Road, Harbin 150069, China; microbiologist_madiha@yahoo.com (M.F.); luoyuzi@caas.cn (Y.L.); 15947055849@163.com (L.Z.); songhao1109@163.com (H.S.); fuyanhui20190301@163.com (Y.F.); liyongfeng@caas.cn (Y.L.); sunyuan@caas.cn (Y.S.); lisu@caas.cn (S.L.); 2 State Key Laboratory of Biocatalysis and Enzyme Engineering, School of Life Sciences, Hubei University, Wuhan 430062, China; pywang@stu.hubu.edu.cn

**Keywords:** classical swine fever virus, genetic typing, molecular characterization, subgenotype 2.1, antigenicity

## Abstract

Classical swine fever (CSF) is a highly contagious disease of swine caused by classical swine fever virus (CSFV). For decades the disease has been controlled in China by a modified live vaccine (C-strain) of genotype 1. The emergent genotype 2 strains have become predominant in China in the past years that are genetically distant from the vaccine strain. Here, we aimed to evaluate the current infectious status of CSF, and for this purpose 24 isolates of CSFV were identified from different areas of China during 2016–2018. Phylogenetic analysis of NS5B, E2 and full genome revealed that the new isolates were clustered into subgenotype 2.1d and 2.1b, while subgenotype 2.1d was predominant. Moreover, E2 and E^rns^ displayed multiple variations in neutralizing epitope regions. Furthermore, the new isolates exhibited capacity to escape C-strain-derived antibody neutralization compared with the Shimen strain (genotype 1). Potential positive selection sites were identified in antigenic regions of E2 and E^rns^, which are related with antibody binding affinity. Recombination events were predicted in the new isolates with vaccine strains in the E2 gene region. In conclusion, the new isolates showed molecular variations and antigenic alterations, which provide evidence for the emergence of vaccine-escaping mutants and emphasize the need of updated strategies for CSF control.

## 1. Introduction

Classical swine fever (CSF) is a highly infectious viral disease of swine notifiable to the World Organization of Animal Health (OIE) due to its devastating impact on the swine industry [1,2]. The disease is characterized by a broad spectrum of clinical signs that range from fever, hemorrhage, erythema, dyspnea, huddling, weakness and cyanosis [3]. CSF can run acute, chronic, or subclinical courses depending on the age of the infected animals and the virulence of the virus [4,5].

CSF is caused by classical swine fever virus (CSFV), a member of the genus *Pestivirus* within the family *Flaviviridae* [6,7]. The viral genome is a single-stranded, positive-sense RNA genome of 12.3 kb that contains one open reading frame (ORF) surrounded by 5′ and 3′ un-translated regions (UTRs) [8]. The ORF is translated into a 3898-AA polyprotein that is later cleaved into individual proteins, including four structural (C, E^rns^, E1, and E2) and eight non-structural proteins (P7, N^pro^, NS2, NS3, NS4A, NS4B, NS5A and NS5B) [8,9]. Of the structural proteins E2 is the most immunogenic and has been shown to induce strong neutralizing antibodies [10]. E2 has also been shown to interact with cell surface receptors that determine host tropism [11,12,13]. E^rns^ is unique because it possesses intrinsic ribonuclease (RNase) activity that can inhibit the production of type I interferons [13,14]. All the three glycoproteins E2, E^rns^ and E1 are involved in virus attachment and entry into target cells. E1-E2 heterodimers are essential for viral entry and infectivity [13,14,15].

CSF is endemic in many parts of the world, including Central and South America, Asia, Eastern Europe, and Africa [4] with recent outbreaks occurring in Japan [16]. Although the disease has been successfully eradicated from domestic pig populations in many countries, CSFV is still prominent in the wild boar population and there is a significant risk of re-emergence of CSFV due to transfers between infected wild boars and domestic pigs [17]. The recent outbreak of CSF in Japan which has been CSFV free for 26 years, has shown the potential for a rapid reintroduction of the virus into a CSF-free country. Three genotypes of CSFV have been reported worldwide with several subgenotypes, including 1.1, 1.2, 1.3, 1.4, 2.1, 2.2, 2.3, 3.1, 3.2, 3.3, and 3.4 [17,18,19,20].

In China, CSF outbreaks continue, despite the wide number of C-strain vaccinated swine holdings [21,22,23]. Four subgenotypes (1.1, 2.1, 2.2, and 2.3) have been reported as circulating strains [20,24,25,26]. In 1940s strains of genotype 1 (known as the Shimen strain) were circulating in China. Thereafter, a C-strain vaccine was developed in 1955 based on the genotype 1 strain and subsequently widely used for vaccination [27]. Since 1980s genotype 2.1 strains have been circulating in China, particularly subgenotype 2.1b strains that have become predominant in the last decade and remain endemic in many regions of China [24,27,28,29,30]. Recently, subgenotype 2.1d strains have been reported in C-strain vaccinated pig herds. Importantly, our group has shown that C-strain vaccination would not provide pathological and virological protection against 2.1d strain. However, the pathogenicity of the 2.1d strain was low when compared to the highly virulent Shimen strain [22].

China is the world’s leading producer of pigs, producing over one-half of all swine in the world [31]. The swine industry in China has invested huge efforts to control CSF through prophylactic vaccination [5], however, the disease is still endemic in many regions of China. Previously we have reported that recent isolates could escape the neutralization of porcine anti-C-strain sera. Therefore, there is urgent need to understand the genetic differences and molecular characteristics of the new isolates of CSFV [17,32].

Here, we identified and characterized the new viral isolates of CSFV circulating in the field of China based on the NS5B and E2 genes. Further, representatives of the new isolates were subjected to virus isolation and full-genome sequencing and characterization.

## 2. Materials and Methods

### 2.1. Clinical Samples and Cells

A total of 126 porcine tissue samples including lymph nodes, tonsils, spleen, lungs, and kidneys were submitted to our lab for diagnosis. The samples were collected by farmers from dead or sick pigs suspected of suffering from CSF from pig farms in different provinces (Heilongjiang, Inner Mongolia, Jilin, Shanghai, Beijing, Wuhan, Shandong, Anhui, Shanxi, Jiangsu and Hebei) of China during 2016–2018. Porcine kidney cells (PK-15) were purchased from the China Center for Type Culture Collection (CCTCC, Wuhan, China) and maintained in Dulbecco’s modified Eagle’s medium (DMEM, Thermo-Fisher Scientific, Carlsbad, CA, USA) supplemented with 10% heat-inactivated fetal bovine serum (FBS) (Sigma-Aldrich, St. Louis, MO, USA), and 1% antibiotic-antimycotic (10,000 IU/mL of penicillin, 10,000 µg/mL of streptomycin and 25 µg/m of amphotericin B (Gibco, Grand Island, NY, USA), and incubated at 37 °C with 5% CO_2_.

### 2.2. Virus Isolation

The tissue samples (1 g, 1 cm^3^) were homogenized and resuspended in a 9-fold amount of DMEM. After two freeze-thaw cycles the tissue homogenates or cultures were centrifuged for 5 min at 6000× *g* (4 °C). The supernatant was collected and further filtered by using 0.45-µm filters (Merck Millipore, Cork, Ireland) and inoculated into the confluent monolayer of PK-15 cells (V/V, 1:10). The cells were incubated at 37 °C and 5% CO_2_ for 72 h. The presence of viral particles was confirmed by PCR and indirect immunofluorescence assay (IFA).

### 2.3. RNA Extraction

Total RNA was extracted from the supernatant of tissue homogenates or cell cultures by TRIzol reagent (Invitrogen, Carlsbad, CA, USA) in accordance with the manufacturer’s instructions. RNA was diluted in 30 µL of DEPC (diethyl pyrocarbonate)-treated water (Biosharp, Hefei, China).

### 2.4. RT-PCR, Cloning, and Sequencing

Initially, partial NS5B gene of CSFV was amplified from extracted RNA by reverse-transcription-polymerase chain reaction (RT-PCR). Briefly, cDNA was synthesized using the reverse transcriptase enzyme (TaKaRa, Dalian, China) along with random nucleotides in a final reaction volume of 50 μL containing 1× buffer, 1 mM of each of the dNTPs, 1 U/μL RRI and 6μL of RNA template. Subsequently, PCR was conducted using *Ex Taq* HS polymerase in accordance with the manufacturer’s instructions. Forward primer CSFV-E2-S (5′-TTGAAGAGGYRGGACAGGT-3′) and reverse primer CSFV-E2-R (5′-TGGTCTTRACTGGRTTGTTRGTC-3′) were used to amplify the complete E2 gene of CSFV (1119 bp). Thirteen pairs of primers were designed by the software SnapGene Viewer (version: 3.2.1) and used to amplify the complete genome of CSFV (Table S1). The PCR reaction conditions for the NS5B gene amplification consisted of an initial denaturation for 5 min at 95 °C, followed by 35 amplification cycles (denaturation, 30 s at 95 °C; annealing, 30 s at 55 °C; extension, 1 min at 72 °C) and a final extension for 10 min at 72 °C. The E2 gene and full-genome amplification conditions were with initial denaturation for 5 min at 95 °C, followed by 35 cycles (denaturation, 1 min at 95 °C; annealing, 1 min at 55 °C; extension, 1 min 45 s at 72 °C) and a final extension for 10 min at 72 °C using a PCR thermal cycler (TaKaRa, Otsu, Shiga, Japan). The amplified DNA was stained with the nucleic acid stain gene SafeView^TM^ (Applied Biological Material, Richmond, BC, Canada) in 1.5% agarose gel and further visualize by Gel Doc XR+ System. The PCR product was purified and cloned into the pMD18-T vector (TaKaRa, Dalian, China) and the mixture was transformed into *E. coli* (DH5α) competent cells. The selected positive colonies were cultured and sent to Comate Bioscience (Jilin, China) for sequencing. 

### 2.5. Immunofluorescence Assay

PK-15 cells were seeded into 96-well plates and 100 TCID_50_ CSFV was inoculated into each well. The cells were incubated at 37 °C with 5% CO_2_ for 48 h, then repeatedly washed for 2-times with PBS and subjected to fix with pre-chilled absolute ethanol at −20 °C for 30 min. Thereafter, the CSFV-anti-E2 monoclonal antibody (1:300 diluted with 5% bovine serum albumin) was used as primary antibody and incubated with cells at 37 °C for 2 h. The cells were washed and incubated at 37 °C for 1 h with anti-mouse IgG conjugated with FITC (Sigma-Aldrich) (diluted 1:100 with 5% BSA). Finally, after washing four times with PBST and 1-time with PBS, 50% glycerol was added into each well. The results were recorded under the fluorescence microscope (Nikon TE200; Tokyo, Japan) and viral titers of the cultures were determined using the Reed and Müench method [33].

### 2.6. Sequence Alignment, Phylogenetic Analysis and Characterization

The new isolates of CSFV were analyzed based on partial NS5B gene (23 isolates), full-length of E2 gene (24 isolates) or complete genome sequences. Multiple sequence alignments were generated using the software Lasergene (Version 7.1) (DNASTAR Inc., Madison, WI, USA) and MEGA6 (Center for Evolutionary Functional Genomics, the Bio Design Institute, Tempe, AZ, USA) [34]. The phylogenetic trees were constructed using the neighbor-joining (NJ) method with 1000 bootstrap replicates and the evolutionary distances were computed using the Jukes-Cantor model [35]. The NS5B and E2 gene sequences of the new isolates (Table 1) along with reference sequences downloaded from GenBank (Table S2) were used for phylogenetic analysis.

The phylogenetic tree of the complete genome of 8 new isolates along with 40 reference sequences (Table S3) was constructed and sequence identity were compared with other nine representative CSFV strains using the Clustal W method of Lasergene (Version 7.1) (Table 2). The genetic variations in E1, E2 and E^rns^ proteins were investigated in detail by CLC Sequence Viewer 8.0, together with other reference isolates of CSFV.

### 2.7. One-Step Growth Curve

The confluent monolayer of PK-15 cells in 24-well culture plates (Corning, Shanghai, China) were infected with CSFV Shimen strain, HL18-494 or NM16-323 at a multiplicity of infection (MOI) of 5 and incubated on ice for 1 h. Thereafter, the cells were washed and inoculums were replaced with pre-warmed fresh DMEM and incubated for 1 h at 37 °C. The extracellular virus was inactivated by low pH citrate buffer (pH = 3.0) according to the previously reported protocols [36]. The cell cultures were harvested at 0, 12, 24, 36, 48, 60, 72, 84, 96, 108 and 120 h post-infection (HPI). After two freeze-thaw cycles the supernatant was centrifuged to remove the cellular debris and stored at −80 °C. The viral infectivity was detected by TCID_50_ according to the Reed and Müench method [33]. Standard deviations (SD) and average values of three independent experiments were calculated. NY, United States

### 2.8. Serum Neutralization Test

The antigenic differences between the new isolates (HL18-494, HL18-490, HL18-416, NM16-323, and NM16-333) and classical Shimen strain were evaluated by the neutralization test. C-strain-vaccinated porcine sera and C-strain-vaccinated and subsequently Shimen strain-challenged sera were tenfold serially diluted (starting from 1/10) with DMEM after heat-inactivated for 30 min at 56 °C (Table 3). Diluted samples were mixed with the equal volume of 200 TCID_50_ of CSFV and incubated at 37 °C for 1 h. The antibody-virus mixtures were then added to the 96-well plates containing PK-15 cells for 48 h at 37 °C. The neutralizing antibody titers were defined as the reciprocal of the highest serum dilution that blocked the infection according to the EU Diagnostic Manual [37]. The inhibition rates of the serum were ranged from 62.75% to 81.6%, as determined by CSFV ELISA kit (IDEXX Laboratories, Schiphol-Rijk, The Netherlands).

### 2.9. Selection Pressure Analysis of E2 and E^rns^

The selection pressure acting on the gene E2 and E^rns^ was evaluated based on the ratio ω between non-synonymous substitutions (dN) and synonymous substitutions (dS). To test the differential selection between the lineages of genotype 1 and genotype 2, we selected 17 CSFV stains encompassing major subgenotypes (2.1a, 2.1b, 2.1c, 2.1d, 1.1, and 1.4) and performed likelihood ratio tests (LRT) of selection pressures on E2 and E^rns^ using the branch-site Model A implemented in PAML [38,39]. The lineage of genotype 2 was treated as the foreground. The null model is M1a with the degree of freedom equals to 2. The sites under positive selection with a posterior probability ≥ 0.7 using empirical Bayes tests [38] were tabulated.

### 2.10. Recombination Analysis

The full-genome alignment of 53 non-redundant CSFV strains was scanned for recombination using seven different algorithms (RDP, GENECONV, Bootscan, MaxChi, Chimaera, SiScan, and 3SEQ) implemented in RDP v4 [40]. The recombination fragment was reported if it was detected by three or more of the methods implemented in RDP4.

### 2.11. Statistical Analysis

Data were analyzed using software GraphPad Prism (version 6.01) (GraphPad Software Inc., San Diego, CA, USA) and expressed as mean ± SD. Differences were considered significant by *p*-value (* *p* < 0.05; ** *p* < 0.01).

## 3. Results

### 3.1. Identification of New CSFV Isolates from Clinical Samples

**Figure 1:**
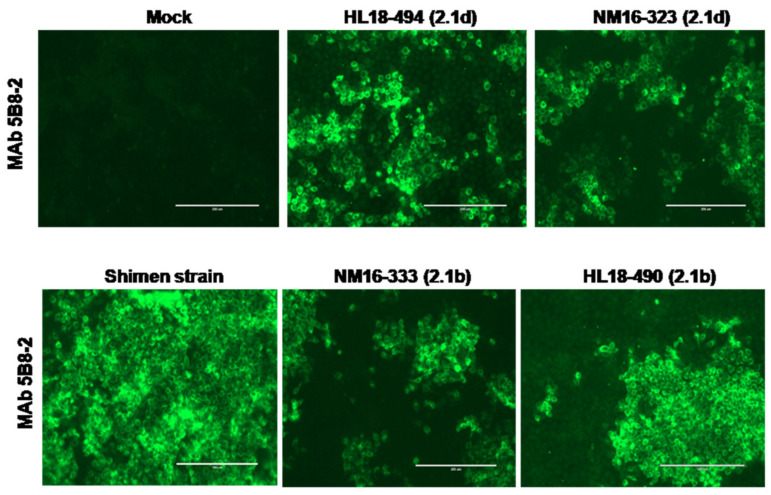
Confirmation of CSFV in PK-15 cells by IFA. The figure represented the reaction of anti-E2 MAb 5B8-2 with Shimen, NM16-323, NM16-333, HL18-494 and HL18-490 strains of CSFV. The scale bar is 200 μm.

**Figure 2:**
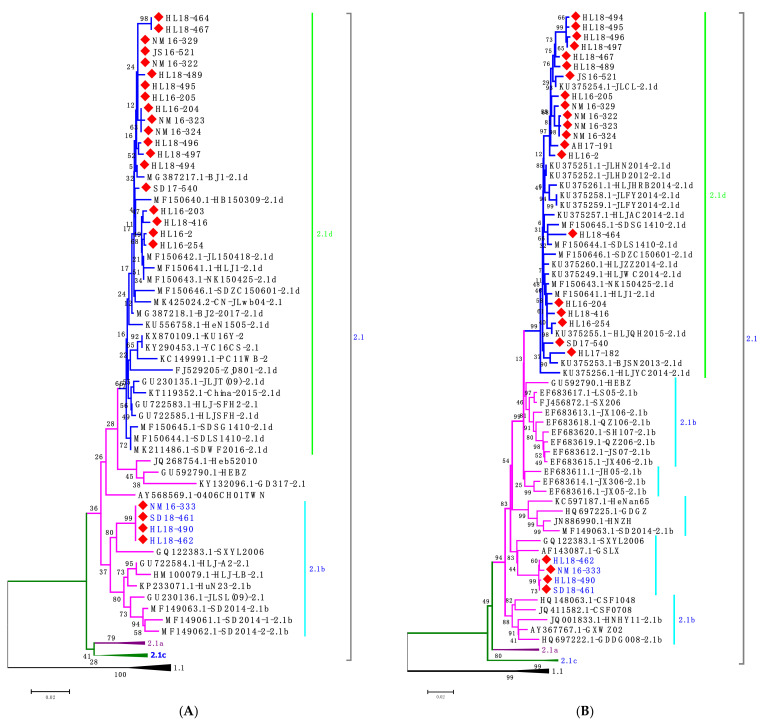
Phylogenetic tree based on nucleotide sequences of NS5B and E2. (**A**) Phylogenetic analysis of the 23 new isolates and 46 reference strains based on partial NS5B gene. (**B**) Phylogenetic analysis of the full-length E2 genes of the 24 new isolates with comparison of 47 reference strains. The trees were constructed using the neighbor-joining (NJ) method, with 1000 bootstrap replicates. The new CSFV isolates were denoted by red diamonds, in which, subgenotype 2.1b isolates were indicated in blue.

In the present study, tissue samples that were collected from CSF-suspected pig farms in different provinces of China were analyzed and 24 samples tested positive for CSFV by RT-PCR. These samples were further processed for virus isolation and confirmed by IFA using Anti-E2 MAb 5B8-2 (Figure 1). The samples were further subjected to amplify NS5B and E2 genes for sequencing to identify different CSFV isolates (Table 1). In total, 69 reference strains were used for phylogenetic analysis of NS5B and E2 genes. The phylogenetic analysis revealed that 19 isolates and 20 isolates were clustered into subgenotype 2.1d based on NS5B and E2 genes, respectively, but four isolates (HL18-490, HL18-462, SD18-461 and NM16-333) were clustered into subgenotype 2.1b (Figure 2).

### 3.2. Full Genome Characterization of the New CSFV Isolates

**Figure 3:**
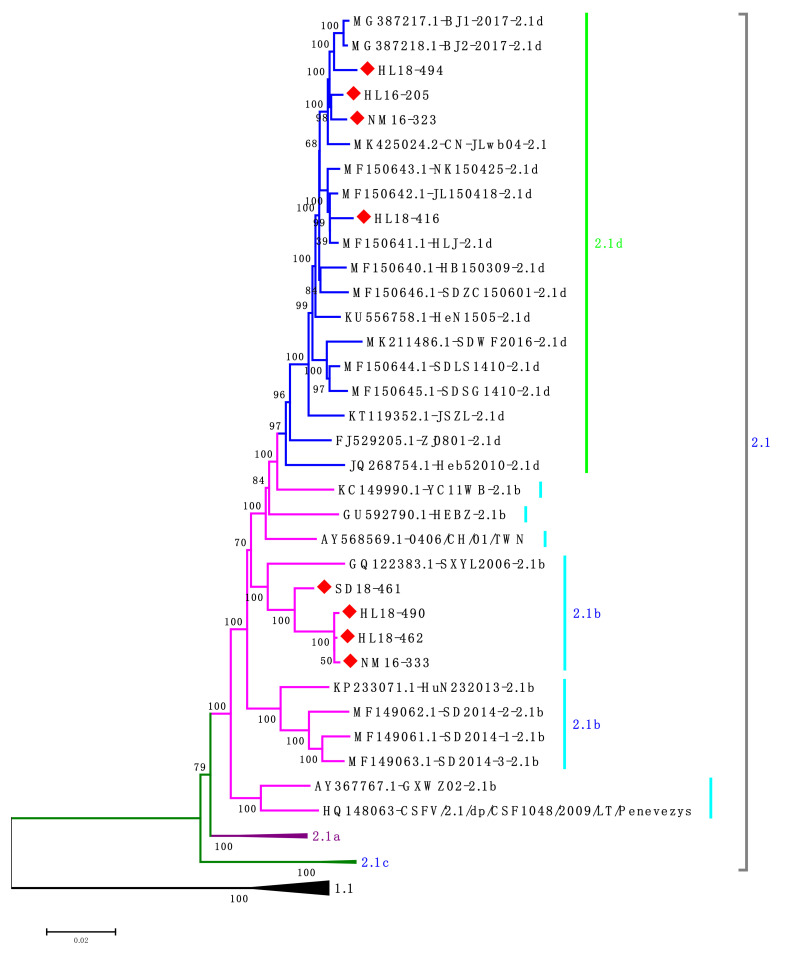
Phylogenetic tree based on the complete genome sequences of CSFV strains using the neighbor-joining (NJ) method, with 1000 bootstrap replicates. All new isolates were indicated by red diamonds, in which the subgenotype 2.1d isolates were indicated in pink while subgenotype 2.1b isolates were indicated in blue.

Based on the phylogenetic analysis of E2 and NS5B genes, four isolates of subgenotype 2.1d and four isolates of subgenotype 2.1b from different regions during 2016–2018 were selected for full-length genome analysis (Table 2). The results demonstrated that four isolates (HL18-494, HL16-205, HL18-416 and NM16-323) were clustered into the subgenotype 2.1d and the other four isolates (HL18-462, HL18-490, NM1633 and SD18-461) were clustered into subgenotype 2.1b (Figure 3). Notably, all CSFV isolated strains were distant from the genotype 1 group and closest to the genotype 2.1 CSFV strains.

Complete nucleotide sequences of the eight new isolates were further compared with nine reference CSFV strains, including Shimen (1.1), C-strain (1.1), Paderborn (2.1a), HEBZ (2.1b), HNSD-2012 (2.1c), JSZL (2.1d), CSFV39 (2.2), Alfort/Tüebingen (2.3), and 94.4/IL/94/TWN (3.4) (Table 3). The eight new isolates shared 95.2%–98.0% and 94.5%–94.9% identity with subgenotype 2.1d and 2.1b strains, respectively. In addition, they shared 87.4%–94.7% sequence identity with other genotype 2 isolates, including 2.1a, 2.1c, 2.2 and 2.3. The new strains shared much lower identities with subgenotype 1.1 (Shimen strain and C-strain, 85.1%–85.2%; 84.5%–84.8%) and subgenotype 3.4 strains (83.4%–83.5%). These results indicate that all new isolates are phylogenetically distant from the vaccine strain.

### 3.3. Genetic Variations in the E2 Protein

**Figure 4:**
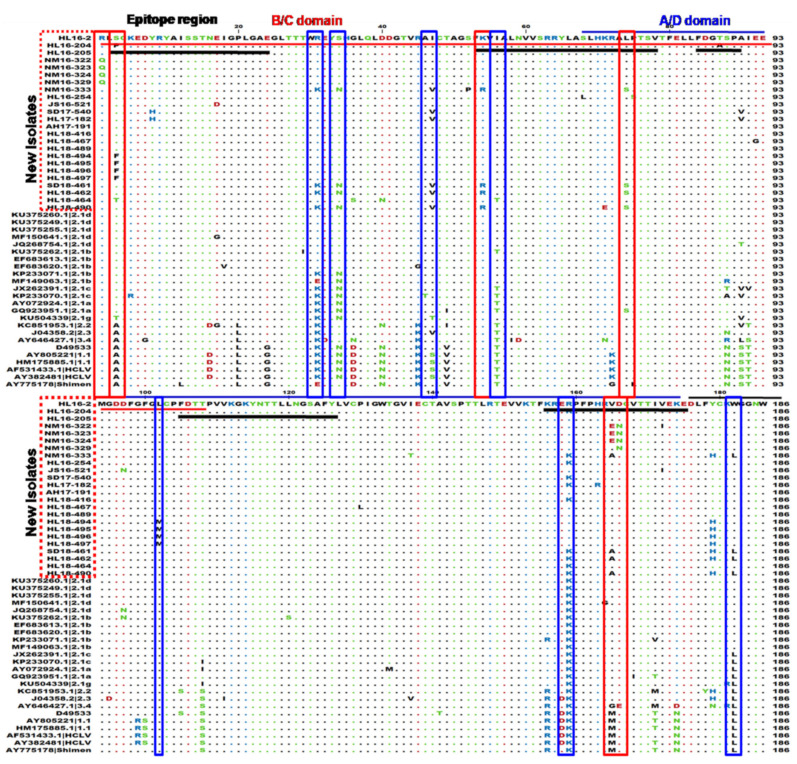

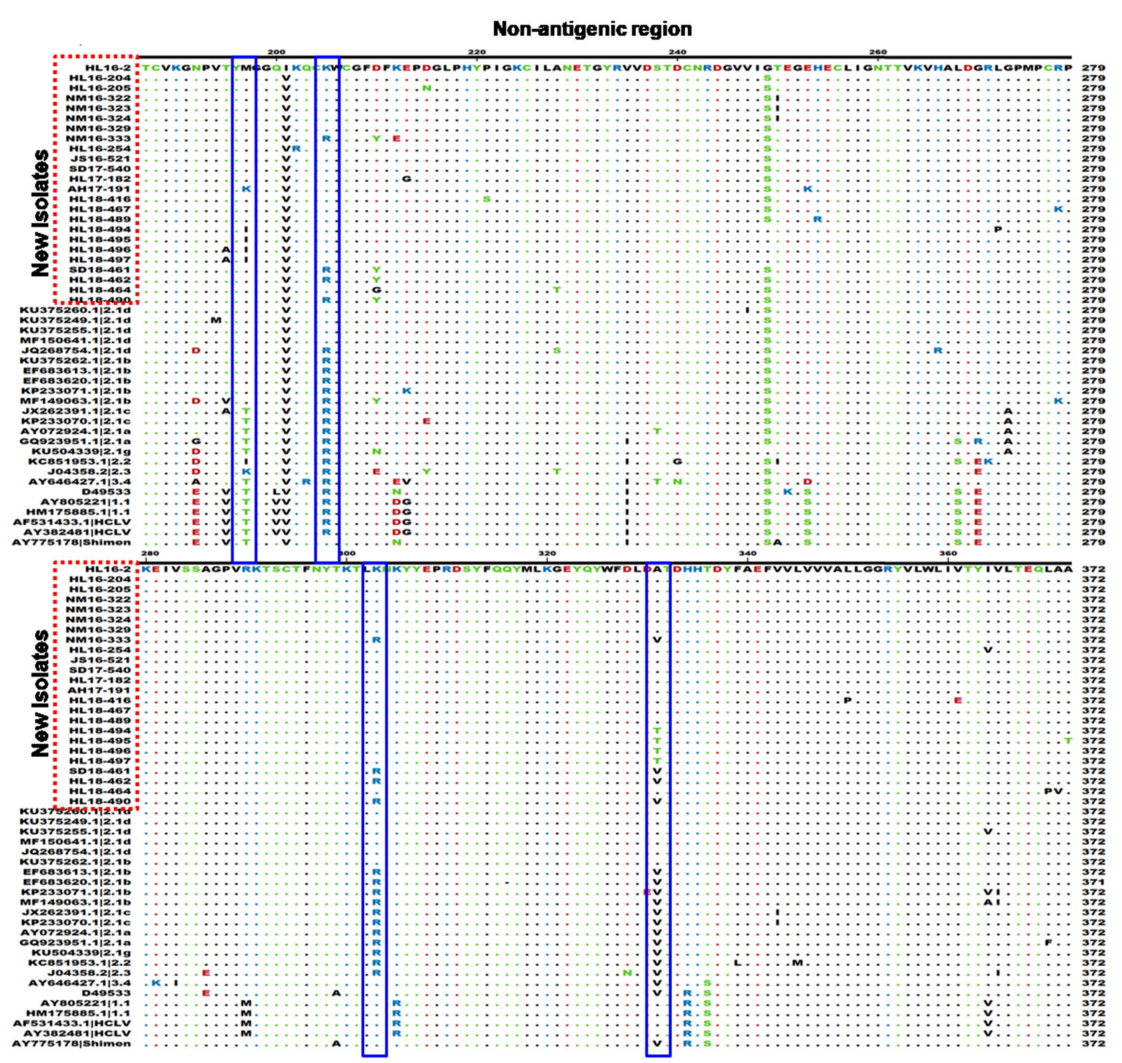
E2 sequence alignment of 24 new CSFV isolates. E2 amino acid sequence alignment of the 24 new CSFV strains isolated in our study, and 24 reference isolates downloaded from NCBI. The B/C domain is indicated by red line, while A/D domain is indicated by blue line and non-antigenic region is indicated by black line. Important epitope regions are also indicated by black line in antigenic regions.

E2 is a highly immunogenic protein, and is a likely protein where mutations could occur that allow the new isolates to escape HCLV vaccination. The amino acid substitutions that were observed in our fully sequenced strains were mostly observed in antigenic regions either B/C or A/D. Alignment results showed that most of the new isolates together with our previously reported subgenotype 2.1d isolates contain variations at various positions 31 (K to R), 34 (N to S), 56 (T to I), 90 (S to A), 159 (K to R), 182 (L to W), 205 (R to K), 303 (R to K) and 331 (V to A), in which the mutation at positions 56 and 159 were located in neutralizing antigenic epitope regions (aa52-76 and aa155-176) corresponding to aa741-765 and 844-865 of the polyprotein, respectively (Figure 4). The four new isolates of subgenotype 2.1b (HL18-490, HL18-462, SD18-461 and NM16-333) contain mutation at positions 54 (K to R), 74 (L to S) and 165 (V to A) in neutralizing epitope regions (aa52-76 and aa155-176) and 47 (I to V). Some new isolates from Inner Mongolia (NM16-322, NM16-323, NM16-324 and NM16-329) contain mutations at positions 165 (V to E) and 166 (D to N) in neutralizing epitope region (aa155-176) corresponding to aa844-865 of the polyprotein. The new isolates (HL18-494, HL18-495, HL18-496 and HL18-497) showed mutation at positions 3 (S to F), 102 (L to N), 197 (M to I), 249 (S to G) and 331 (V to T).

### 3.4. Genetic Variations in the E1 and E^rns^ Protein

**Figure 5:**
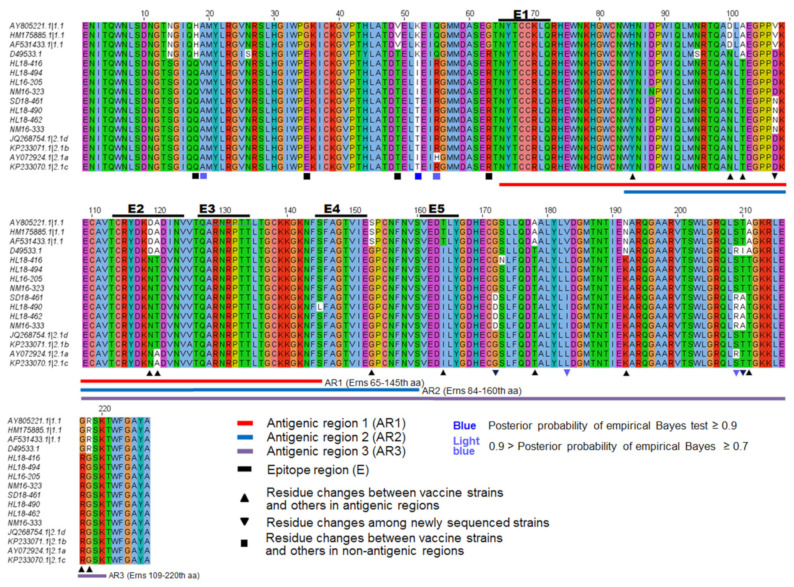
Sequence alignment of amino acids of E^rns^ from 8 new CSFV strains isolated in our study, and 8 reference isolates downloaded from NCBI. Red, blue and purple lines indicate the antigenic regions AR1, AR2 and AR3, respectively. E^rns^ protein residue changes between vaccine strains and others in antigenic regions are indicated by (▲) symbol, residue changes among newly sequenced strains are indicated by (▼) symbols and those between vaccine strains and others in non-antigenic regions are indicated by (■) symbol.

E^rns^ protein contains three antigenic regions AR1, AR2 and AR3 [15,41]. Alignment results showed the four new isolates of subgenotype 2.1d (HL18-416, HL18-494, HL16-205 and NM16-323) contain mutations at positions 52 (T to I), and another four new isolates of subgenotype 2.1b (HL18-490, HL18-462, SD18-461 and NM16-333) contain mutations at positions 107 (D to N), 172 (G to D), 209 (S to R) and 210 (T to A) compared with previous reference isolates (Figure 5).

In E1 protein, five conserved cysteine residues were observed at positions 5, 20, 24, 94 and 123. In addition, we found the four new isolates of subgenotype 2.1d (HL18-494, HL16-205, NM16-323 and HL18-416) contain a mutation at position 27 (K to T or I) and other four new isolates of subgenotype 2.1b (HL18-490, HL18-462, SD18-461 and NM16-333) contain a mutation at 146 (V to I) (Figure S1).

### 3.5. Selection Pressure Analysis

We used likelihood ratio tests (LRTs) of branch-site models implemented in PAML to identify the potential positive selection in the antigenic proteins E2 and E^rns^ in the lineage of genotype 2 in comparison with the lineage of genotype 1. The tests showed that a small proportion of sites in E2 and E^rns^ have a significantly higher ω value (ω_2_ ~ 4.0, *p <* 10^−5^) in the branch leading to the lineage of genotype 2, indicating strong positive selection on the sites (Table 4). Specifically, the positive selection was identified on six sites in E2 and five sites in E^rns^ (posterior probabilities ≥ 0.7) (Table 5). Among them, three sites of E2 (3, 90, and 165) and two sites of E^rns^ (183 and 209) located in the antigenic regions of the two proteins, respectively (Figure S2). The different selective pressures on the sites in the lineage of genotype 2 indicated the adaptive evolution of the antigenic proteins during interaction with host immune systems.

### 3.6. Recombination Analysis

**Figure 6:**
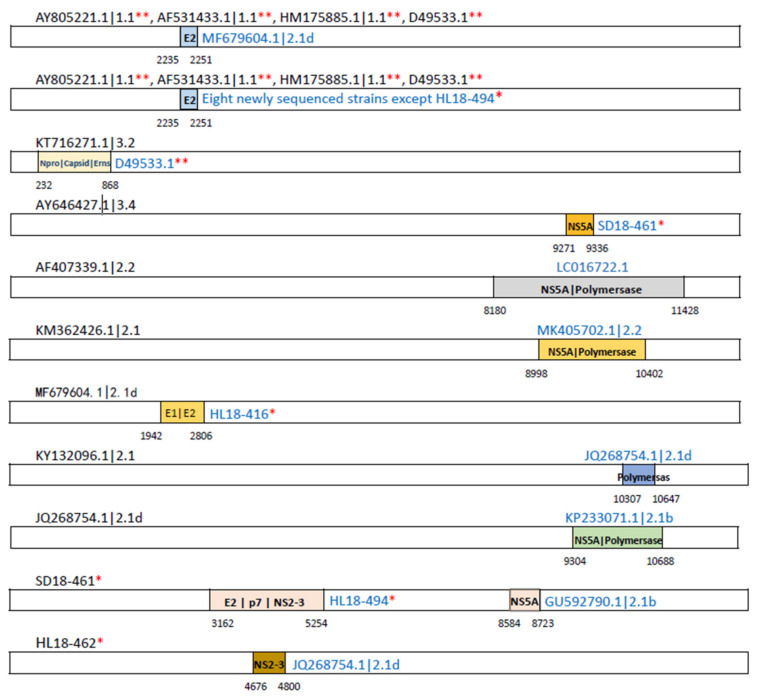
Schematic diagram showing the potential recombination events among different CSFV isolates. The genomic locations and breakpoints of the potential recombining segments are schematically indicated with colored frames. *, the new isolates in this study; **, C-strain vaccine.

Recombination is an important mechanism for the rapid evolution of viruses, which may change the genomic makeup or phylogenetic relationships. To gain insights into recombination events in the CSFV populations, the recombination profile analysis was performed for 53 non-redundant CSFV strains including the eight new field isolates. We identified frequent recombination events potentially occurring in the CSFV-encoded genes among the CSFV strains and most of the events were identified to contain recombining break points between the strains of genotype 2 (Figure 6 and Table S4). Notably, a fragment of NS5A in the new isolate SD18-461 showed the break points of recombining from a genotype 2.1b strain with accession number GU592790; a fragment of NS2 in the new isolate HL18-462 contains the break points of recombining from a genotype 2.1d strain with accession number JQ268754. Interestingly, the vaccine strains (genotype 1.1) are shown to contain a small fragment in the E2 gene with break points of recombining from the new field isolates or an isolate of genotype 2.1d strain with accession number MF679604 (Figure 6). However, examination of the protein sequences reveals a high level of conservation of the amino acids in this region among CSFV, implying negligible functional influences of this recombining region. It indicated that the vaccine strains might have recombined with the ancestral strains of genotype 2.1.

### 3.7. Growth Kinetics of the CSFV Isolates

**Figure 7:**
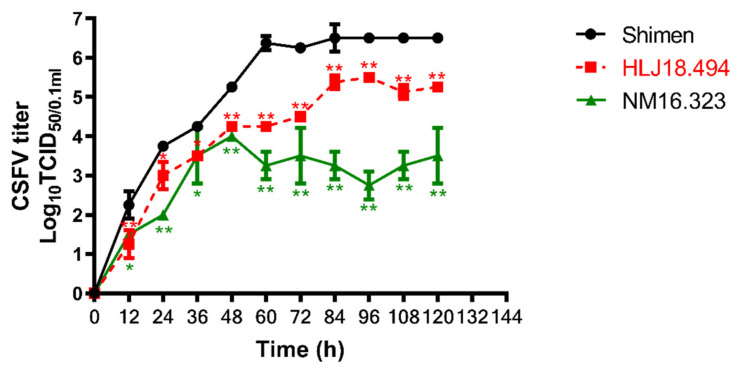
One-step growth curve analysis. Growth kinetics of the CSFV HL18-494, NM16-323 and Shimen strains was depicted by one-step growth curve. An MOI of 5 was used to infect PK-15 cells. The cell cultures were harvested at various time points and TCID_50_ was calculated. The mean values with standard deviations of three independent experiments were calculated. *, *p* < 0.05; **, *p* < 0.01, compared with the Shimen strain.

Considering the subgenotype 2.1d isolates are the most predominant and the sequence variations are observed among the new isolates, we selected the new isolates HL18-494 and NM16-323 to investigate the replication characteristics of the CSFV subgenotype 2.1d isolates in PK-15 cells. As shown in Figure 7, HL18-494 exhibited lower virus titers at 12, 24, 36, 48, 60, 72, 84, 96, 108 and 120 HPI (*p* < 0.05) compared with the reference virus Shimen strain, but relatively higher than the NM16-323 strain.

### 3.8. Antigenicity of the CSFV Isolates

E2 alignment and characterization showed the mutations in antigenic domains, including neutralizing epitope regions. Therefore, it is necessary to investigate the antigenicity of the new isolates. Neutralization test showed that the neutralizing titers of the porcine anti-C-strain sera against the five new isolates (HL18-416, HL18-494, NM16-323 NM16-333 and HL18-490) were found lower than those of the Shimen strain (genotype 1.1). Moreover, the investigation showed that there was no remarkable difference among the neutralizing titers of all the new isolates (Table 6).

## 4. Discussion

Genotyping based on partial NS5B and full-length E2 sequences has been an accepted standard for CSFV classification, but the whole-genome sequence of CSFV could provide a better resolution and a more reliable classification criterion [14,42,43]. Hence, we performed complete genome analysis along with NS5B and E2 genotyping in order to depict a more clear epidemic situation of CSFV in China. These isolates were obtained from C-strain-vaccinated pig farms located in different regions of China. Most of the new isolates were recognized as subgenotype 2.1d and 16.7% (4/24) of the new isolates were clustered into subgenotype 2.1b, indicating that recently subgenotype 2.1d was predominant in China.

Next, we attempted to analyze the molecular characterization of the new isolates. The envelop glycoprotein E2 is an important element for viral entry and induce immunogenic reactions in pigs [44,45]. The architecture of E2 protein is divided into carboxyl-terminal half and N-terminal half. The N-terminal half contain two antigenic units, B/C (aa690-797 or aa1-108) and D/A (aa757-863 or aa68-174) [9,10,44,46]. Here, specific aa substitutions were identified on E2 protein of the new isolates at multiple positions 54 (K to R), 56 (T to I), 74 (L to S), 159 (K to R), 165 (V to E) and 166 (D to N), which were corresponding to aa positions 743, 745, 763, 848, 854 and 855 of polyprotein, respectively. These variations are located in neutralizing epitope regions, aa741-760, 753-765 and 844-865 of the polyprotein that have been previously reported [47,48,49].

According to a previous report, variations within antigenic domains of E2, D705N, L709, G173E, N723S and S779A had markedly affected the neutralization efficacy of heterologous strains [45]. Here, we found same mutations along with other variations and confirmed that the porcine anti-C-strain sera neutralized the new isolated CSFV strains less efficiently compared with the highly virulent Shimen strain (genotype 1). Such antigenic variation may explain why subgroup 2.1 CSFV strains persist in China despite the wide use of vaccine C-strain. Antibody selection may be the possible reason for the conversion of viral strain from genotype 1 to 2 [45]. In short, the results indicated that the investigated porcine sera were not effectively neutralizing all the selected new isolates. The variations in the immune trigger points, especially in the B/C domain, are linked with viral escape and require further in-depth study.

The neutralization escape of the new isolates took our concern towards vaccination pressure and recombination analysis. The differential neutralization between the new isolates of genotype 2 and the vaccine strains of genotype 1 prompted us to perform the selection analysis to decipher the potential selection pressures acting on the genes in the new isolates. In the present study, several amino acid replacements were found at the B/C domain in E2 when CSFV reference strains were compared. Among the detected sites under positive selection, three sites of E2 (3, 90, and 165) and two sites of E^rns^ (183 and 209) are located in the antigenic domains of the two proteins, respectively. Specifically, the sites 90 and 165 in E2 (corresponding to the position of 779 and 854 in the polyprotein of CSFV) have been shown to affect the antibody binding and were proposed to be responsible for antigenic specificity [44]. It supports our observation of decreased neutralizing efficacy for the new isolates against the C-strain-derived antibodies in comparison with the Shimen strain. Therefore, these positive selection sites may represent the adaptive changes of the antigenic proteins during the interaction with host immune systems that ultimately lead to immune escape in the lineage of genotype 2. Several studies have also demonstrated the level of positive selection on the codon sites of the E2 (49, 72, 75 and 200) and E^rns^ (208) proteins, or six sites (34, 36, 49, 72, 87 and 88) in the E2 protein of CSFV [25,50,51]. Another positively selected site 192 in E2 is located in a functional domain 182-262, which was demonstrated to interact with beta-actin involved in early replication of CSFV [52]. The change from glutamate to asparagine at the residue 192 of E2 could possibly be related with the lower titers of the new isolates of genotype 2 in the viral growth study. Nevertheless, the precise functional impacts of the sites require further investigation.

In this study, we analyzed full-length sequences of CSFV isolates and found that homologous recombination happens frequently among CSFV strains. There are only few reports on recombination analysis of CSFV [53,54]. We identified break points for recombination in the genes encoding C, E1, E2, E^rns^, N^pro^, NS5A, and p7 proteins. There are possibilities of coexistence of different types of livestock husbandry methods, stamping-out vaccination strategies, and unrestricted movement of commercial herds. All of these factors may have had influences on the spread of virus, alterations and evolution [53]. According to a previous study, vaccination is an important causative factor for the emergence of new CSFV isolates through recombination and these emerging isolates may pose a greater danger to pig health and industry due to unpredictable phenotypic characteristics [55].

In summary, the new CSFV isolates have undergone frequent variations and antigenic alterations. Moreover, detailed analysis exposed the events of vaccination pressure and recombination among vaccine type and the non-vaccine type groups. The study exposed the myth that evolutionary changes in CSFV might be due to disproportionate usage of the C-strain vaccines in endemic areas. This underscores the need to develop mitigation vaccination strategies to minimize the substantial risk associated with the emergence of vaccine escaping mutants.

## Figures and Tables

**Table 1:** New isolated strains of CSFV from China in this study based on NS5B and E2 genes.

No	Isolate	GenBank Accession for NS5B Sequence	GenBank Accession for E2 Sequence	Year	Area
1	AH17-191	-	MT777567	2017	Anhui
2	HL16-02	MT777591	MT777568	2016	Heilongjiang
3	HL16-203	MW367226	-	2016	Heilongjiang
4	HL16-204	MT777592	MT777569	2016	Heilongjiang
5	HL16-205	MT777593	MT777570	2016	Heilongjiang
6	HL16-254	MT777594	MT777571	2016	Heilongjiang
7	HL17-182	-	MT777572	2017	Heilongjiang
8	HL18-416	MT777595	MT777573	2018	Heilongjiang
9	HL18-462	MT777596	MT777574	2018	Heilongjiang
10	HL18-464	MT777597	MT777575	2018	Heilongjiang
11	HL18-467	MT777598	MT777576	2018	Heilongjiang
12	HL18-489	MT777599	MT777577	2018	Heilongjiang
13	HL18-490	MT777600	MT777578	2018	Heilongjiang
14	HL18-494	MT777601	MT777579	2018	Heilongjiang
15	HL18-495	MT777602	MT777580	2018	Heilongjiang
16	HL18-496	MT777603	MT777581	2018	Heilongjiang
17	HL18-497	MT777604	MT777582	2018	Heilongjiang
18	JS16-521	MT777605	MT777583	2016	Jiangsu
19	NM16-322	MT777606	MT777584	2016	Inner Mongolia
20	NM16-323	MT777607	MT777585	2016	Inner Mongolia
21	NM16-324	MT777608	MT777586	2016	Inner Mongolia
22	NM16-329	MT777609	MT777587	2016	Inner Mongolia
23	NM16-333	MT777610	MT777588	2016	Inner Mongolia
24	SD17-540	MT777611	MT777590	2018	Shandong
25	SD18-461	MT777612	MT777589	2018	Shandong

**Table 2:** New isolated strains of CSFV from China in this study based on the full-genome.

No	Strain	Origin	Accession
1	HL18-462	Heilongjiang	MT799517
2	HL18-416	Heilongjiang	MT799514
3	HL18-490	Heilongjiang	MT799518
4	HL18-494	Heilongjiang	MT799515
5	SD18-461	Shandong	MT799516
6	HL16-205	Heilongjiang	MT799513
7	NM16-323	Inner Mongolia	MT799519
8	NM16-333	Inner Mongolia	MT799520

**Table 3:** Nucleotide sequence identities of the new isolates with nine reference strains based on the full genome.

New Isolated Strains	Shimen (1.1)	HCLV (1.1)	Paderborn (2.1a)	HEBZ (2.1b)	HNSD-2012 (2.1c)	JSZL (2.1d)	CSFV39 (2.2)	Alfort/Tüebingen (2.3)	94.4/IL/94/TWN(3.4)
	N.A%	N.A%	N.A%	N.A%	N.A%	N.A%	N.A%	N.A%	N.A%
HL16-205	85.1	84.6	94.2	94.8	92.3	98.0	87.6	89.9	83.5
HL18-416	85.2	84.6	94.1	94.5	92.1	97.8	87.5	89.6	83.5
HL18-462	85.1	84.7	94.7	94.9	92.6	95.2	87.7	90.1	83.6
HL18-490	85.1	84.7	94.6	94.8	92.6	95.2	87.6	90.1	83.5
HL18-494	85.1	84.5	93.9	94.5	92.0	97.6	87.4	89.6	83.4
NM16-323	85.1	84.5	94.2	94.7	92.2	97.9	87.4	89.7	83.5
NM16-333	85.1	84.7	94.6	94.8	92.6	95.2	87.6	90.0	83.6
SD18-461	85.2	84.8	94.6	94.8	92.6	95.9	87.6	901	83.6

**Table 4:** The likelihood ratio test results of Model A in PAML for the E2 and E^rns^ genes.

Tested Genes	Model A					Model A vs. Null	
	p_1_	p_2a_	p_2b_	ω_2a_	ω_2b_	LRT ^a^	*p*-Value
E2	0.1687	0.1257	0.03145	4.0945	4.0945	20.50	1.25 × 10^−9^
E^rns^	0.2164	0.1464	0.05437	4.3713	4.3713	12.33	4.40 × 10^−6^

^a^ LRT is the likelihood ratio test statistic calculated as 2Δl with l the log-likelihood for each model. The null model is the Model 1a.

**Table 5:** The sites of E2 and E^rns^ under potential positive selection in the likelihood ratio test of Model A.

Gene	Location in the Segment	Location in the Full Gene	Posterior Pr.(ω > 1)
E2	3	692	0.705
	90	779	0.878
	165	854	0.709
	179	868	0.828
	182	871	0.928
	192	881	0.710
E^rns^	19	286	0.834
	52	319	0.926
	55	322	0.813
	183	450	0.817
	209	476	0.728

The sites with the posterior probability *p* ≥ 0.7 are shown. The positions refer to the codon positions in the multiple sequence alignment of each gene.

**Table 6:** Neutralization titers of anti-C-strain sera against various CSFV isolates.

No	Antisera ID	Information of the Sera	Shimen	HL18-416	HL18-494	NM16-323	NM16-333	HL18-490
1	SPF	/	<10	<10	<10	<10	<10	<10
2	81	C-strain-vaccinated	80	<10	20	20	30	20
3	83	C-strain-vaccinated	40	10	20	30	20	10
5	468	C-strain-vaccinated/Shimen-challenged	160	<10	20	15	40	20
6	79	C-strain-vaccinated/Shimen-challenged	320	10	40	60	80	40

## Data Availability

The genomic sequences of the 8 new isolates have been deposited in the NCBI GenBank with accession numbers MT799513-MT799520. The nucleotide sequences of NS5B from 23 new isolates were deposited in the NCBI GenBank with accession numbers MW367226 and MT777591-MT777612, and E2 from 24 new isolates with accession numbers MT777567-MT777590.

## Supplementary Materials

The following are available online at https://www.mdpi.com/article/10.3390/v13040664/s1, Figure S1: E1 sequence alignment of the 8 new CSFV isolates. Figure S2: E2 sequence alignment of the 8 new CSFV isolates. Table S1: Primers for full genome analysis used in the study, Table S2: Reference strains used for NS5B and E2 gene analysis. Table S3: Reference CSFV strains used for complete genome analysis. Table S4: Genetic recombination in the CSFV strains.
