# Supplementary material for: Genotyping and Molecular Characterization of Classical Swine Fever Virus Isolated in China during 2016–2018
Source: Viruses. 2021 Apr 12;13(4):664. doi: 10.3390/v13040664 (PMC8069065; doi:10.3390/v13040664)
Supplement: Supplementary file 1 [file viruses-13-00664-s001.zip › Supplementary Files/Figures S1-S2.docx]

**
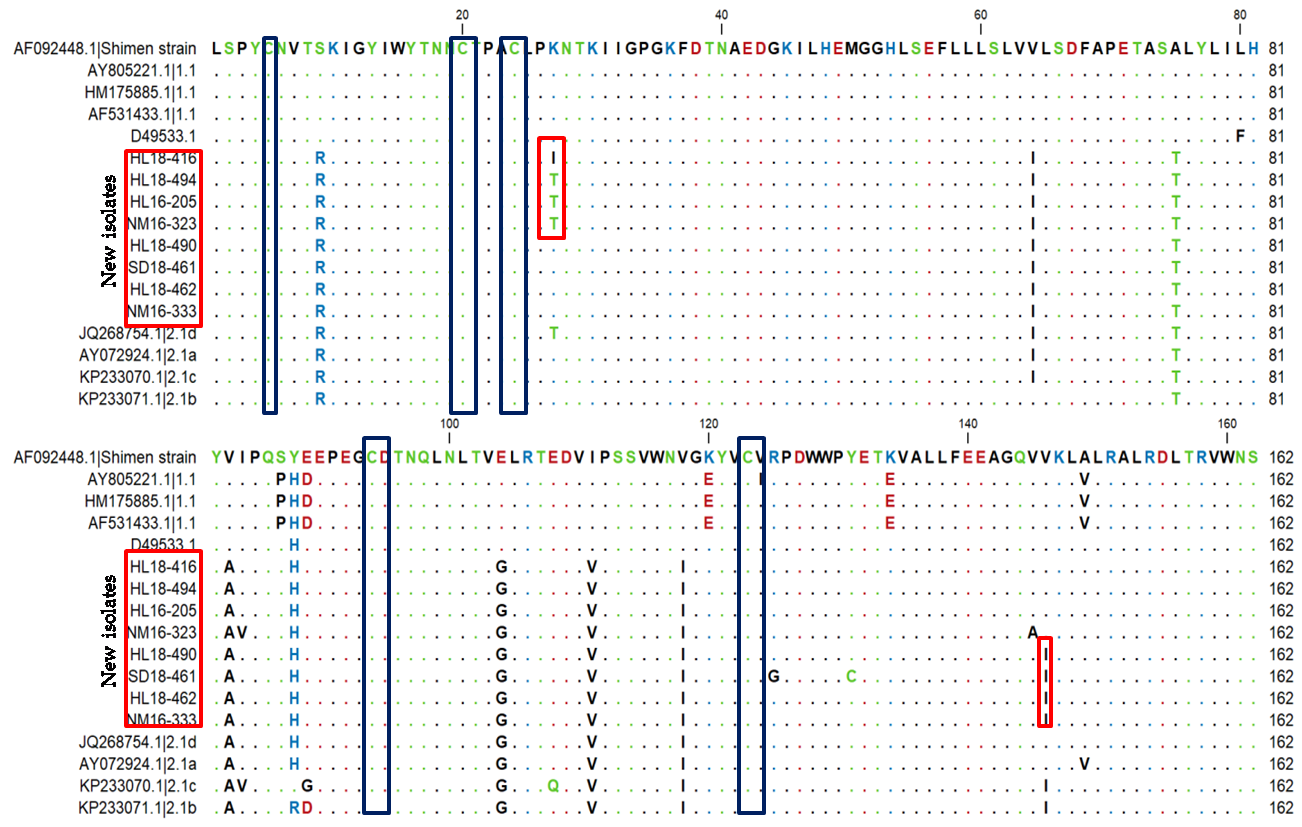
**

**Figure S1.** E1 amino acid sequence alignment of the 8 new CSFV strains isolated in our study, and 8 reference isolates downloaded from NCBI. Blue box highlighted the conserved cysteine residues and red box highlighted the difference among new isolates.

**Figure S2.** Sequence alignment of E2 of 8 new full-length genome isolates. E2 amino acid sequence alignment of the 8 new CSFV strains isolated in our study, and 7 reference isolates downloaded from NCBI. The B/C domain is indicated by red line, while A/D domain is indicated by blue line.
