# Supplementary material for: Genotyping and Molecular Characterization of Classical Swine Fever Virus Isolated in China during 2016–2018
Source: Viruses. 2021 Apr 12;13(4):664. doi: 10.3390/v13040664 (PMC8069065; doi:10.3390/v13040664)
Supplement: Supplementary file 1 [file viruses-13-00664-s001.zip › Supplementary Files/Tables S1-S4.docx]

**Table S1. Primers used in the study for full genome analysis.**

| \| **No** \| **Primer name** \| **Primer’s sequence** \| **bp** \| \| --- \| --- \| --- \| --- \| \| 1 \| PF.1 (1-21) \| GTATACGAGGTTAGCTCATCC \| 21 \| \| PR.1 (988-1008) \| CTCTACCACAATCGTAGCATC \| 21 \| \| 2 \| PF.2 (790-810) \| CTGTTGAAGTTAGCCAAGAGG \| 21 \| \| PR.2 (1964-1984) \| CGTCTTCTGCGTTGGTGTCAA \| 21 \| \| 3 \| PF.3 (1854-1874) \| CCTGTCACCTTACTGCAATGT \| 21 \| \| PR.3 (2880-2900) \| GTCTTCACCACTTCTGTTCTC \| 21 \| \| 4 \| PF.4 (2692-2712) \| GATGGGACTAGTCCAGCGATT \| 21 \| \| PR.4 (3535-3555) \| GGCAGCAAGTTGCTCTGTTAG \| 21 \| \| 5 \| PF.5 (3467-3487) \| TTGTCTTGGTGGTAGTGGCAC \| 21 \| \| PR.5 (4716-4736) \| GCCACTACTTCATTCCTCACC \| 21 \| \| 6 \| PF.6 (4601-4621) \| GACTAGTGGCAGCCTTAATTG \| 21 \| \| PR.6 (5600-5620) \| CCTTGAGGTTCTTGAGGTCAA \| 21 \| \| 7 \| PF.7 (5557-5579) \| GGAGAATTCACTTGCGTAACAGC \| 21 \| \| PR.7 (6612-6632) \| CCAATGGTGACAGCCATTCTT \| 21 \| \| 8 \| PF.8 (6453-6471) \| GGTAGTCACATCGCAGTCC \| 19 \| \| PR.8 (7362-7382) \| GCCAACTCCTTCAATTCAGTC \| 21 \| \| 9 \| PF.9 (7286-7306) \| CAGTCGAAGATCACAGGCTAG \| 21 \| \| PR.9 (8369-8389) \| CCAGTAGTTCCACGGCCTCAA \| 21 \| \| 10 \| PF.10 (8022-8044) \| GGTTAGTGCGGCTATGGAGAT \| 21 \| \| PR.10 (9104-9124) \| GCCAGGTTGTAACCGTAATGG \| 21 \| \| 11 \| PF. 11 (8749-8773) \| CCAGTGATAAGAATGGAAGGACACG \| 24 \| \| PR.11(10024-10044) \| TTGAGCCAGTTGGTAGGCTGA \| 21 \| \| 12 \| PF.12 (9966-9986) \| TGAAGAGCTCCTGCAACAGTG \| 21 \| \| PR.12 (11319-11339) \| GGCTTACCAGCTTCATATAGG \| 21 \| \| 13 \| PF.13 (11054-11075) \| CTATGCACATGTCAGAAGTACC \| 22 \| \| PR.13 (12259-12279) \| ACCTTAGTCCAACTATGGACG \| 21 \| |
| --- | --- | --- | --- | --- | --- | --- | --- | --- | --- | --- | --- | --- | --- | --- | --- | --- | --- | --- | --- | --- | --- | --- | --- | --- | --- | --- | --- | --- | --- | --- | --- | --- | --- | --- | --- | --- | --- | --- | --- | --- | --- | --- | --- | --- | --- | --- | --- | --- | --- | --- | --- | --- | --- | --- | --- | --- | --- | --- | --- | --- | --- | --- | --- | --- | --- | --- | --- | --- | --- | --- | --- | --- | --- | --- | --- | --- | --- | --- | --- | --- | --- | --- | --- | --- | --- | --- | --- | --- | --- | --- | --- | --- | --- | --- | --- |

**Table S2. Reference strains used for NS5B and E2 gene analysis.**

| **No** | **Virus strain** | **Year** | **Origin** | **Genotype** | **GenBank accession** |
| --- | --- | --- | --- | --- | --- |
| 1 | HCLV | 2003 | China | 1.1 | AY382481 |
| 2 | Riems | 2003 | Switzerland | 1.1 | AY259122 |
| 3 | Koslov | 2013 | Denmark | 1.1 | KF977608 |
| 4 | Shimen | 2004 | China | 1.1 | AY775178 |
| 5 | C/HVRI | 2004 | China | 1.1 | AY805221 |
| 6 | 96TD | 2004 | Taiwan | 2.1a | AY554397 |
| 7 | SXCDK | 2009 | China | 2.1a | GQ923951 |
| 8 | JS07 | 2016 | China | 2.1b | EF683612 |
| 9 | LS05 | 2016 | China | 2.1b | EF683617 |
| 10 | SH107 | 2011 | China | 2.1b | EF683620 |
| 11 | SD2014 | 2014 | China | 2.1b | MF149063 |
| 12 | 0406CH01TWN | 2005 | Taiwan | 2.1 | AY568569 |
| 13 | HNLY | 2009 | China | 2.1c | JX262391 |
| 14 | GXF292013 | 2013 | China | 2.1c | KP233070 |
| 15 | HLJWC2014 | 2014 | China | 2.1d | KU375249 |
| 16 | BJSN2013 | 2013 | China | 2.1d | KU375253 |
| 17 | HLJAC2014 | 2013 | China | 2.1d | KU375257 |
| 18 | HLJZZ2014 | 2014 | China | 2.1d | KU375260 |
| 19 | HLJ1 | 2013 | China | 2.1d | MF150641 |
| 20 | NK150425 | 2013 | China | 2.1d | MF150643 |
| 21 | SDSG1410 | 2013 | China | 2.1d | MF150645 |
| 22 | JSZL | 2015 | China | 2.1d | KT119352 |
| 23 | SDLS1410 | 2014 | China | 2.1d | MF150644 |
| 24 | SDZC150601 | 2015 | China | 2.1d | MF150646 |
| 25 | BJ1 | 2017 | China | 2.1d | MG387217 |
| 26 | JLJT(09) | 2009 | China | 2.1d | GU230135 |
| 27 | HLJ-SFH | 2010 | China | 2.1d | GU722585 |
| 28 | Zj0801 | 2008 | China | 2.1d | FJ529205 |
| 29 | JL150418 | 2015 | China | 2.1d | MF150642 |
| 30 | SD2014-1 | 2018 | China | 2.1b | MF149061 |
| 31 | SD2014-2 | 2014 | China | 2.1b | MF149062 |
| 32 | SD2014-3 | 2014 | China | 2.1b | MF149063 |
| 33 | BJ2-2017 | 2017 | China | 2.1d | MG387218 |
| 34 | SDWF-2016 | 2016 | China | 2.1d | MK211486 |
| 35 | JXNC01-2015 | 2015 | China | 2.1b | KX064281 |
| 36 | HeN1505 | 2015 | China | 2.1d | KU556758 |
| 37 | HuN23/2013 | 2013 | China | 2.1b | KP233071 |
| 38 | C-ZJ-2008 | 2008 | China | 1.1 | HM175885 |
| 39 | HNLY-2011 | 2011 | China | 2.1c | JX262391 |
| 40 | Koslov | 2013 | China | 1.1 | KF977608 |
| 41 | GXF29/2013 | 2013 | China | 2.1c | KP233070 |
| 42 | HB150309 | 2015 | China | 2.1d | MF150640 |
| 43 | GXWZ02 | 2003 | China | 1.1 | AY367767 |
| 44 | JX1-06 | 2008 | China | 2.1b | EF683613 |
| 45 | JX3-06 | 2016 | China | 2.1b | EF683614 |
| 46 | JX4-06 | 2008 | China | 2.1b | EF683615 |
| 47 | JX-05 | 2008 | China | 2.1b | EF683616 |
| 48 | QZ1-06 | 2007 | China | 2.1b | EF683618 |
| 49 | QZ2-06 | 2007 | China | 2.1b | EF683619 |
| 50 | SH1-07 | 2008 | China | 2.1b | EF683620 |
| 51 | SXYL2006 | 2006 | China | 2.1b | GQ122383 |
| 52 | HEBZ | 2009 | China | 2.1b | GU592790 |
| 53 | CSF1048 | 2009 | China | 2.1 | HQ148063 |
| 54 | GDDG | 2008 | China | 2.1 | HQ697222 |
| 55 | GDGZ | 2009 | China | 2.1 | HQ697225 |
| 56 | HNZH | 2011 | China | 2.1 | JN886990 |
| 57 | HNHY11 | 2011 | China | 2.1b | JQ001833 |
| 58 | CSF0708 | 2000 | China | 2.1 | JQ411582 |
| 59 | CSFV/2.1/dp/HeNan65/2012/Henan | 2012 | China | 2.1 | KC597187 |
| 60 | JLHN2014 | 2014 | China | 2.1d | KU375251 |
| 61 | JLHD2012 | 2012 | China | 2.1d | KU375252 |
| 62 | JLCL2015 | 2015 | China | 2.1d | KU375254 |
| 63 | HLJQH2015 | 2015 | China | 2.1d | KU375255 |
| 64 | HLJYC2014 | 2014 | China | 2.1d | KU375256 |
| 65 | HLJAC2014 | 2014 | China | 2.1d | KU375257 |
| 66 | JLFY2014-1 | 2014 | China | 2.1d | KU375258 |
| 67 | JLFY2014-2 | 2014 | China | 2.1d | KU375259 |
| 68 | HLJZZ2014 | 2014 | China | 2.1d | KU375260 |
| 69 | HLJHRB2014 | 2014 | China | 2.1d | KU375261 |

**Table S3. Reference strains used for complete genome analysis.**

| **No** | **Strain** | **Year** | **Origin** | **Genotype** | **GenBank accession** |
| --- | --- | --- | --- | --- | --- |
| 1 | HLJ1 | 2015 | China | 2.1d | MF150641 |
| 2 | NK150425 | 2017 | China | 2.1d | MF150643 |
| 3 | SDLS1410 | 2017 | China | 2.1d | MF150644 |
| 4 | SDZC150601 | 2015 | China | 2.1d | MF150646 |
| 5 | SD2014-13 | 2014 | China | 2.1b | MF149063 |
| 6 | JSZL | 2014 | China | 2.1d | KT119352 |
| 7 | BJ1-2017 | 2017 | China | 2.1d | MG387217 |
| 8 | HCLV | 2002 | China | 1.1 | AF531433 |
| 9 | Paderborn | 2002 | Denmark | 2.1a | AY072924 |
| 10 | Riems | 2003 | Switzerland | 1.1 | AY259122 |
| 11 | HCLV | 2003 | China | 1.1 | AY382481 |
| 12 | 96TD | 2005 | Taiwan | 2.1a | AY554397 |
| 13 | 0406/CH/01/TWN | 2005 | Taiwan | 2 | AY568569 |
| 14 | C/HVRI | 2006 | China | 1.1 | AY805221 |
| 15 | GPE- | 1995 | Japan | 1.1 | D49533 |
| 16 | Zj0801 | 1995 | Japan | 2.1d | FJ529205 |
| 17 | SXCDK | 2009 | China | 2.1a | GQ923951 |
| 18 | C-ZJ-2008 | 2008 | China | 1.1 | HM175885 |
| 19 | Heb52010 | 2010 | China | 2.1d | JQ268754 |
| 20 | HNLY-2011 | 2011 | China | 2.1c | JX262391 |
| 21 | Koslov | 2013 | Denmark | 1.1 | KF977608 |
| 22 | Shimen/HVRI | 2004 | China | 1.1 | AY775178 |
| 23 | cF114 | 2001 | China | 1.1 | AF333000 |
| 24 | JL1(06) | 2006 | China | 1.1 | EU497410 |
| 25 | JL150418 | 2015 | China | 2.1d | MF150642 |
| 26 | SDSG1410 | 2014 | China | 2.1d | MF150645 |
| 27 | SXYL2006 | 2006 | China | 2.1b | GQ122383 |
| 28 | GXF29/2013 | 2013 | China | 2.1c | KP233070 |
| 29 | HuN23/2013 | 2013 | China | 2.1b | KP233071 |
| 30 | HeN1505 | 2015 | China | 2.1d | KU556758 |
| 31 | SD2014-1 | 2014 | China | 2.1b | MF149061 |
| 32 | SD2014-2 | 2014 | China | 2.1b | MF149062 |
| 33 | HB150309 | 2015 | China | 2.1d | MF150640 |
| 34 | SDWF-2016 | 2016 | China | 2.1d | MK211486 |
| 35 | CN-JLw04 | 2018 | China | 2.1 | MK425024 |
| 36 | BJ2-2017 | 2017 | China | 2.1d | MG387218 |
| 37 | YC11WB | 2013 | South Korea | 2.1b | KC149990 |
| 38 | CSFV/2.1/dp/CSF1048/2009/LT/Penevezys | 2009 | Lithuania | 2.1 | HQ148063 |
| 39 | GXWZ02 | 2003 | China | 2.1b | AY367767 |

**Table S4. Genetic recombination in CSFV strains.**

| **Recombinant strain** | **Parental strain^a^** | **Recombinant**  **area** | **Related**  **protein**  E2 | |  |
| --- | --- | --- | --- | --- | --- |
| AY805221.1\|1.1** | L49347.1\|3.4 (84.9%); MF679604.1\|2.1d (100%) | 2235~2251 | | E2 | |
| AF531433.1\|1.1** | L49347.1\|3.4 (85%); MF679604.1\|2.1d (100%) | 2235~2251 | | E2 | |
| HM175885.1\|1.1** | L49347.1\|3.4 (84.9%); MF679604.1\|2.1d (100%) | 2235~2251 | | E2 | |
| D49533.1** | L49347.1\|3.4 (85.5%); MF679604.1\|2.1d (100%) | 2235~2251 | | E2 | |
| AY805221.1\|1.1** | HL18-490 (96%); HL18-416 (96%); HL18-462 (96%); NM16-333 (96%); SD18-461 (96%); HL16-205 (96%); NM16-323 (96%) | 2235~2251 | | E2 | |
| KT716271.1\|3.2 | KF669877.1\|3.2 (96.1%); D49533.1** (98%) | 232~868 | | N^pro^, Capsid, E^rns^ | |
| AY646427.1\|3.4 | KX576461.1\|1.4 (85.1%); SD18-461* (97%) | 9271~9336 | | NS5A | |
| AF407339.1\|2.2 | KC533776.2\|2.2 (96.1%); LC016722.1 (97.7%) | 8180~11428 | | NS5A, Polymersase | |
| KM362426.1\|2.1 | KP233071.1\|2.1b (91.7%); MK405702.1\|2.2 (96.9%) | 8998~10402 | | NS5A, Polymersase | |
| MF679604.1\|2.1d | Unknown (GQ122383.1\|2.1b); HL18-416* (97.9%) | 1942~2806 | | E1, E2 | |
| KY132096.1\|2.1 | HL18-494* (94.2%); JQ268754.1\|2.1d (99.3%) | 10307~10647 | | Polymersase | |
| JQ268754.1\|2.1d | KC149990.1\|2.1b (96.6%); KP233071.1\|2.1b (98.4%) | 9304~10688 | | NS5A, Polymersase | |
| SD18-461* | HL18-490* (99%); HL18-494*(98.9%) | 3162~5254 | | E2, p7, NS2-3 | |
| SD18-461* | GQ122383.1\|2.1b (96.3%); GU592790.1\|2.1b (100%) | 8584~8723 | | NS5A | |
| HL18-462* | HL18-490*(99.8%); JQ268754.1\|2.1d (100%) | 4676~4800 | | NS2-3 | |
